# Aberrant Expression of Novel Cytokine IL-38 and Regulatory T Lymphocytes in Childhood Asthma

**DOI:** 10.3390/molecules21070933

**Published:** 2016-07-18

**Authors:** Man Chu, Ida M.T. Chu, Edmund C.M. Yung, Christopher W.K. Lam, Ting F. Leung, Gary W.K. Wong, Chun K. Wong

**Affiliations:** 1 Department of Chemical Pathology, The Chinese University of Hong Kong, Prince of Wales Hospital, Shatin, NT, Hong Kong, China; shaanxichuman@gmail.com (M.C.); idachu1219@gmail.com (I.M.T.C.); 2 Shenzhen Research Institute, The Chinese University of Hong Kong, 518057 Shenzhen, China; 3 Department of Paediatrics, The Chinese University of Hong Kong, Prince of Wales Hospital, Shatin, NT, Hong Kong, China; eyung@cuhk.edu.hk (E.C.M.Y.); tfleung@cuhk.edu.hk (T.F.L.); 4 State Key Laboratory of Quality Research in Chinese Medicine, Macau Institute for Applied Research in Medicine and Health, Macau University of Science and Technology, Taipa, Macau, China; wklam@must.edu.mo; 5 Institute of Chinese Medicine and State Key Laboratory of Phytochemistry and Plant Resources in West China, The Chinese University of Hong Kong, Hong Kong, China

**Keywords:** childhood asthma, cytokines, IL-38, regulatory T lymphocytes

## Abstract

We investigated the expression of novel anti-inflammatory interleukin (IL)-38 and regulatory T (Treg) lymphocytes in childhood asthma patients. The protein and mRNA expression level of IL-38, periostin, peripheral CD4^+^CD25^+^CD134^+^ T lymphocytes as well as CD4^+^CD25^high^FoxP3^+^ and CD4^+^CD25^high^CD127^−^ Treg lymphocytes from 40 asthmatic patients and 20 normal control (NC) subjects were studied using ELISA, qPCR and flow cytometry. Serum and supernatant cytokines/chemokines were determined by multiplex assay. Serum IL-38, IL-5, IL-17, IL-6, interferon-γ, periostin, IL-1β and IL-13 concentrations were significantly higher in asthmatic patients with or without steroid treatment than those in controls (all *p* < 0.05). The percentages of both CD4^+^CD25^high^FoxP3^+^ and CD4^+^CD25^high^CD127^−^ Treg lymphocytes were markedly decreased in asthmatic patients with and without steroid treatment than those in controls (all *p* < 0.05). The elevated IL-38 concentration negatively correlated with the percentage of Treg lymphocytes in asthmatic patients with high level (>40 ng/mL) of periostin (*p* < 0.05). Although the comparable mRNA levels of IL-38 and its receptor IL-36R were found between patients and controls, the mRNA level of IL-38 positively correlated with IL-36R and negatively correlated with IL-10 in all asthmatic patients (both *p* < 0.05). The percentage of CD4^+^CD25^+^CD134^+^ activated T lymphocytes was also significantly higher in asthmatic patients with steroid treatment than those in controls (*p* < 0.05). This cross-sectional study demonstrated that the overexpression of circulating IL-38 may play a role in the immunopathogenesis in asthma.

## 1. Introduction

Asthma is a common inflammatory disease that can cause coughing, wheezing, chest tightness and breathlessness. The activation and infiltration of T lymphocytes, granulocyte eosinophils, basophils, type 2 innate lymphoid cells and mast cells into the airway submucosa play critical roles in the inflammatory process of asthma [1]. Currently, it affects all ages of people in the world, especially children, with the prevalence of childhood asthma reaching up to 30% [2].

The predominant phenotype of childhood asthma is allergic asthma, characterized by circulating allergen-specific IgE antibodies [3]. Allergies against food allergens and inhalant allergens are mediated by an aberrant innate and adaptive immune response. T cells are clearly relevant for adaptive immune regulation, yet the Th1/Th2 paradigm is oversimplified and does not explain the complicated cascade of immunological events from initiation to progression and exacerbation of the allergic responses [4]. Regulatory T (Treg) lymphocytes, the anti-inflammatory T cell subset, have been involved in maintenance and regulation of immune response to infectious organisms in childhood asthma [5]. Human Treg cells were first characterized as CD4^+^CD25^high^ T cells and subsequently confirmed the transcription factor FoxP3 as a specific marker [6]. Positive correlation between CD4^+^CD25^+^CD127^−^ and CD4^+^CD25^high^FoxP3^+^ has been reported in healthy subjects [7] and, these two forms of Treg lymphocytes have been universally accepted as the reliable Treg phenotypes. It has been reported that Treg lymphocytes were deficient in blood of children with asthmatic risk [8], and were increased in neonates from farming mothers with low risk for asthma [9]. Treg lymphocytes could inhibit proliferation and cytokines production by allergen-responsive T cells and exert potential immune suppressive role in asthma [10].

Dysregulation of a number of cytokines has been shown to relate with the immunopathogenesis of allergic asthma [11]. Apart from predominant Th2 signature cytokines IL-13, IL-4 and IL-5, the critical role of IL-6, interferon (IFN)-γ, IL-25, IL-33 and thymic stromal lymphopoietin (TSLP) have been implicated in asthma [12,13,14]. IL-38 is the newest interleukin cytokine which binds with IL-36 receptor, comprising a novel IL-36 subfamily cytokine together with IL-36α, IL-36β, IL-36γ and IL-36 receptor antagonist (IL-36Ra) [15]. Similar to other IL-36 subfamily cytokines, IL-38 is expressed mostly in the skin, although proliferating B cells of the tonsil also express IL-38 [16]. Currently, the biological function of IL-38 remains unclear due to its low expression. However, the anti-inflammatory effect of IL-38 was observed in peripheral blood mononuclear cells (PBMC) culture via the reduced production of IL-17 and IL-22, similar to the functions of IL-36Ra, the natural inhibitor of IL-36R [17]. Moreover, the silenced IL-38-treated PBMC produced up to 30-fold more of the pro-inflammatory mediators IL-6, CCL2 and APRIL (a proliferation-inducing ligand) than control-transfected cells upon toll-like receptor (TLR) agonist stimulation [18]. In the same study, the overexpression of circulating IL-38 in SLE patients has been observed [19]. Besides, significant increased levels of plasma IL-38 and IL-38 gene expression in PBMCs were reported in ST-segment elevation myocardial infarction (STEMI) patients, and IL-38 levels were positively correlated with inflammatory C-reactive protein (CRP), cardiac troponin I (cTNI), and N-terminal of the prohormone brain natriuretic peptide (NT-proBNP) and negatively correlated with left ventricular ejection fraction in STEMI patients [19].

Elevated expression of eosinophilic airway inflammation biomarker periostin has been found in asthmatic mice and bronchial epithelial cells of asthmatic patients [20,21]. Periostin is a secreted matricellular protein associated with fibrosis, and its expression is upregulated by Th2 cytokine IL-4 and IL-13 in bronchial epithelial cells and bronchial fibroblasts [22,23]. Besdes, it has been reported that highly activated CD4^+^CD25^+^CD134^+^ T cells that served as effector cells might be involved in inflammatory disease [24,25]. To further elucidate the immunopathological mechanism(s) mediating inflammation in childhood asthma, we investigated the expression and the relationship of IL-38 and Treg Lymphocytes as well as periostin and activiated T lymphocytes in paediatric patients with asthma.

## 2. Results and Discussion

### 2.1. Asthmatic Patients and Control Subjects

The clinical characteristics of the study population are summarized in Table 1. Twenty non-steroid-treated asthmatic patients, 20 steroid-treated asthmatic patients, and 20 control subjects were enrolled in this study, with their mean ± SD ages at evaluation being 7.3 ± 0.6, 7.5 ± 0.6, and 7.8 ± 0.9 years, respectively (*p* > 0.05). Eosinophils (%) but not basophils (%) were found to be significantly higher in asthmatic patients with or without steroid treatment compared to control subjects (both *p* < 0.001). Most asthmatic patients were found to have specific IgE against at least one of the following inhaled allergens including mixed house dust mite Dp1, house dust mite Df1, cockroaches and cat dander. None of the control subjects presented with specific IgE against the above allergens.

### 2.2. Serum Levels of IL-38, Periostin and Several Common Cytokines

**Figure 1:**
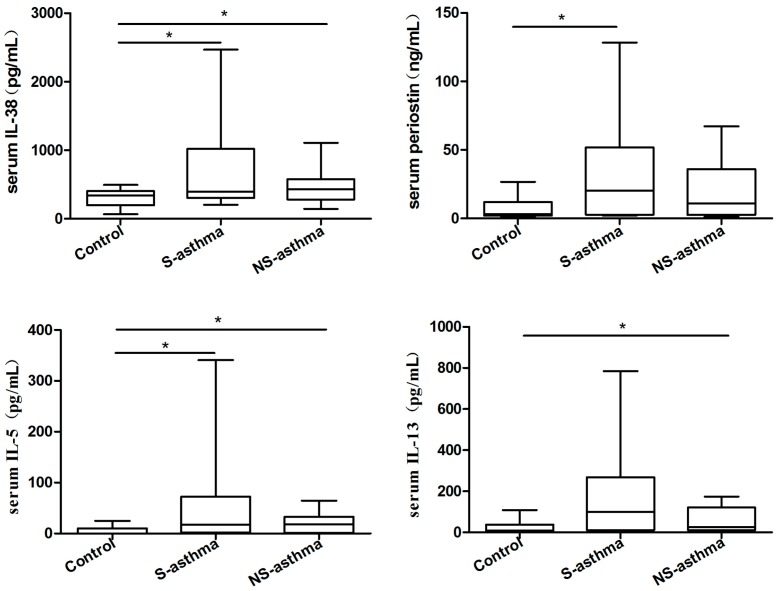

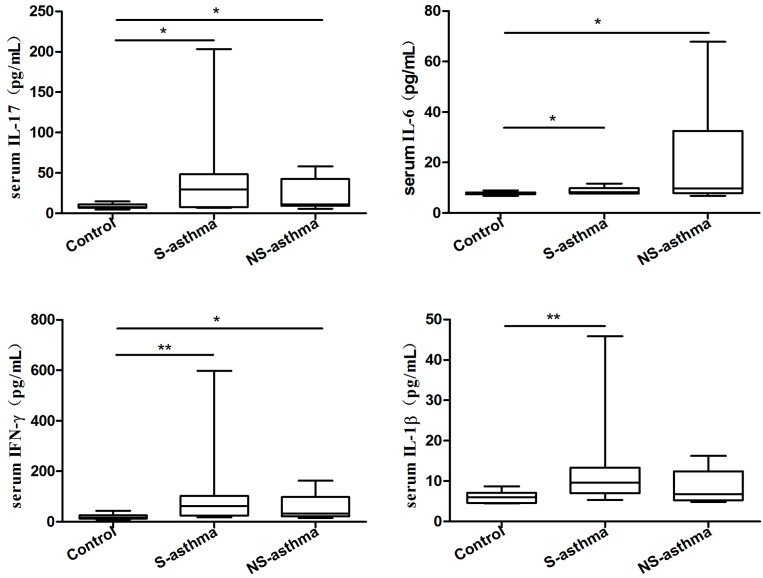
Comparison of serum concentrations of IL-38, periostin, IL-5, IL-13, IL-17, IL-6, IFN-γ and IL-1β between asthmatic patients with or without steroid treatment, and control subjects. Serum IL-38, periostin, IL-5, IL-13, IL-17, IL-6, IFN-γ and IL-1β were measured using ELISA and multiplex assay kit. Results are presented as box and whisker plots with median (interquartile range). Statistical significances were indicated by * *p* < 0.05 and ** *p* < 0.01 (Mann-Whitney U test). S-asthma: steroid-treated asthmatic patients, NS-asthma: non-steroid-treated asthmatic patients, control: control subjects.

There were detectable IL-38, periostin, IL-5, IL-13, IL-17, IL-6, IFN-γ, TNF-α and IL-1β present in the serum of both control subjects and asthmatic patients. As illustrated in Figure 1, serum IL-38, IL-5, IL-17, IL-6 and IFN-γ concentrations were significantly higher in both asthmatic patients with or without steroid treatment than those in controls (all *p* < 0.05). Serum eosinophilic airway inflammation biomarker periostin and IL-1β concentrations were significantly higher in steroid treated asthmatic patients than that in controls while serum asthma-related IL-13 concentrations were significantly higher in non-steroid treated asthmatic patients than that in controls (all *p* < 0.05). Since steroid-treated patients usually have higher disease severity, it is reasonable that periostin and IL-1β concentrations were significantly higher in steroid treated asthmatic patients. Serum Th2 cytokine IL-4 was barely detectable, and TNF-α levels showed no significant difference between control subjects and asthmatic patients (*p* > 0.05).

### 2.3. Circulating Regulatory T Lymphocytes

**Figure 2:**
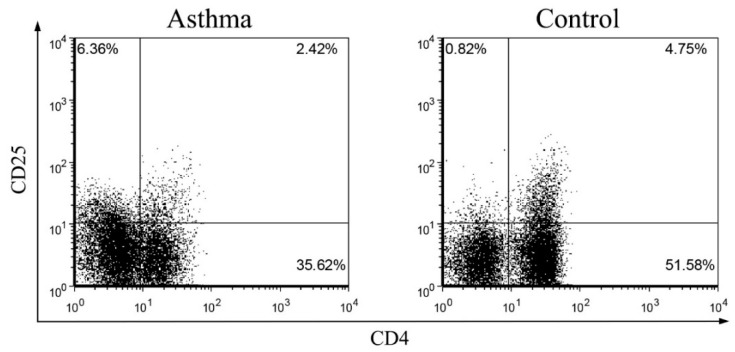

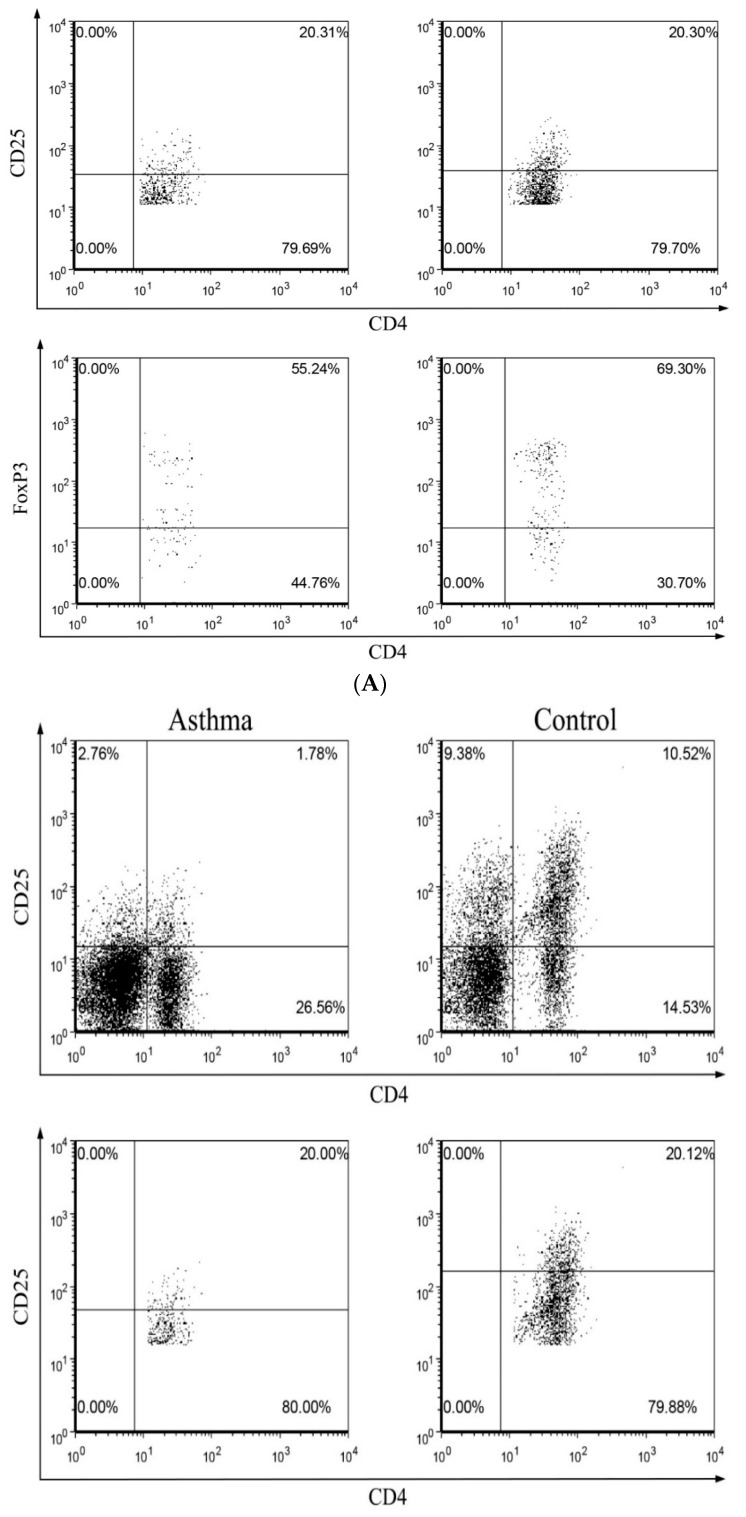

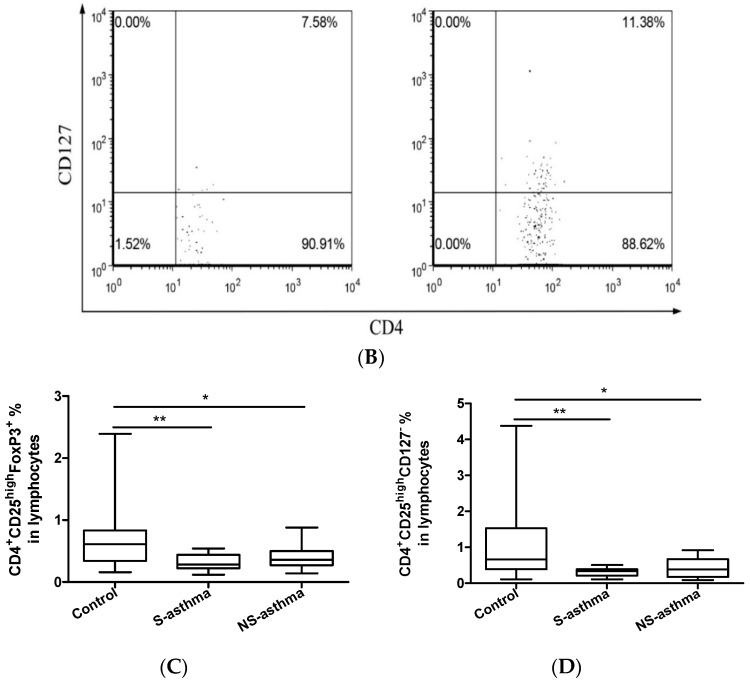
Comparison of circulating CD4^+^CD25^high^FoxP3^+^ and CD4^+^CD25^high^CD127^−^ Treg lymphocytes percentages between asthmatic patients with or without steroid treatment, and control subjects. Representative dot plots and gating strategy are shown for the (**A**) CD4^+^CD25^high^FoxP3^+^ and (**B**) CD4^+^CD25^high^CD127^−^ Treg lymphocytes. Briefly, the CD4^+^CD25^+^ cells were gated from total lymphocytes, and then the FoxP3^+^ or CD127^−^ cells were gated from the top 20% high CD25^+^ cells of CD4^+^CD25^+^ cells; (**C**,**D**) The proportion of circulating Treg lymphocytes in total lymphocytes from asthmatic patients with or without steroid treatment, and control subjects were determined by flow cytometry. Results are presented as box and whisker plots with median (interquartile range). Statistical significances were indicated by * *p* < 0.05 and ** *p* < 0.01 (Mann-Whitney U test). S-asthma: steroid-treated asthmatic patients, NS-asthma: non-steroid-treated asthmatic patients, control: control subjects.

Healthy immune maturation is dependent on anti-inflammatory T cell subsets, namely Treg lymphocytes. The representative dot plots of CD4^+^CD25^high^Foxp3^+^ and CD4^+^CD25^high^CD127^−^ Treg lymphocytes were shown in Figure 2A,B. Briefly, the CD4^+^CD25^+^ cells were gated from total lymphocytes, and then the FoxP3^+^ or CD127^−^ cells were gated from the top 20% high CD25^+^ cells of CD4^+^CD25^+^ cells. In control group, CD4^+^CD25^high^Foxp3^+^ Treg lymphocytes composed [0.6 (0.3–0.8) %] of lymphocytes while in steroid-treated and non-steroid-treated asthmatic patients, corresponding proportions were [0.3 (0.2–0.4) %] and [0.4 (0.3–0.5) %], respectively (Figure 2C).

Similarly, in control group, CD4^+^CD25^high^CD127^−^ Treg lymphocytes composed [0.7 (0.4–1.5) %] of lymphocytes while in steroid-treated and non-steroid-treated asthmatic patients, corresponding proportions were [0.3 (0.2–0.4) %] and [0.4 (0.2–0.8) %], respectively (Figure 2D). The frequencies of circulating CD4^+^CD25^high^Foxp3^+^ and CD4^+^CD25^high^CD127^−^ Treg lymphocytes of total lymphocytes were significantly decreased in both asthmatic patients with or without steroid treatment compared with controls (Figure 2C,D, all *p* < 0.05).

### 2.4. Quantitative Analysis of the mRNA Expression of IL-38

**Figure 3:**
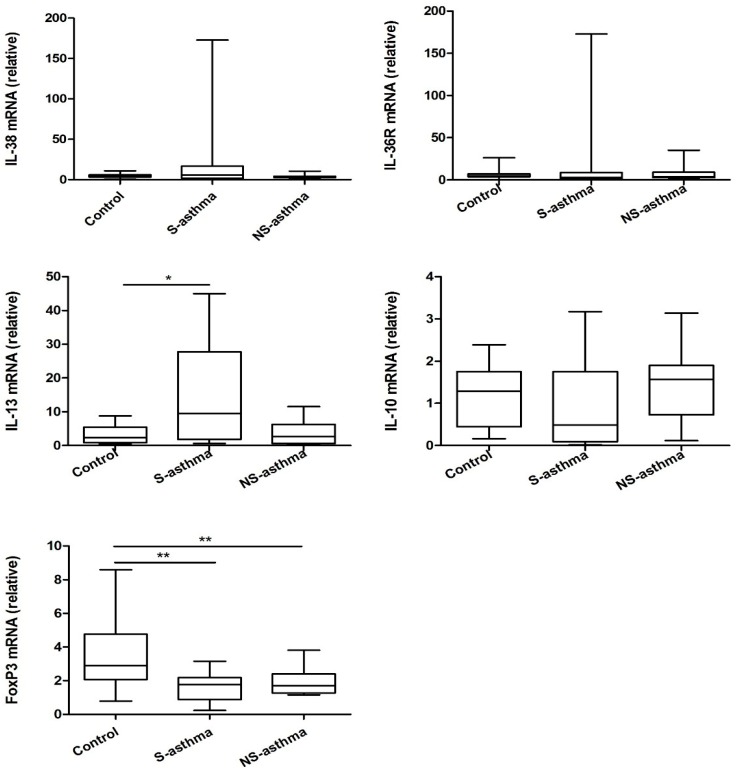
The differences of mRNA expression of IL-38, IL-36R, IL-13, IL-10 and FoxP3 between asthmatic patients and controls. PBMC was purified and total RNA was extracted, reverse transcribed and analyzed by real time PCR. Results are presented as box and whisker plots with median (interquartile range). Statistical significances were indicated by * *p* < 0.05 and ** *p* < 0.01 (Mann-Whitney U test). S-asthma: steroid-treated asthmatic patients; NS-asthma: non-steroid-treated asthmatic patients, control: control subjects.

Given that patients with asthma expressed higher level of IL-38 and lower level of Treg lymphocytes, the mRNA expression level of IL-38, FoxP3 and IL-36 receptor as well as cytokines from PBMC have been analyzed. As shown in Figure 3, for mRNA expression levels of IL-38, IL-36R and IL-10, there were no significant differences in both asthmatic patients with or without steroid treatment compared with controls (all *p* > 0.05). However, the mRNA level of asthma cytokine IL-13 was significantly increased in steroid treated asthmatic patients (*p* < 0.05). Notably, in both asthmatic patients with or without steroid treatment group, the mRNA level of FoxP3 was markedly decreased compared that in control group (both *p* < 0.05, Figure 3).

### 2.5. Correlation among Different Immunological Parameters in Asthmatic Patients

**Figure 4:**
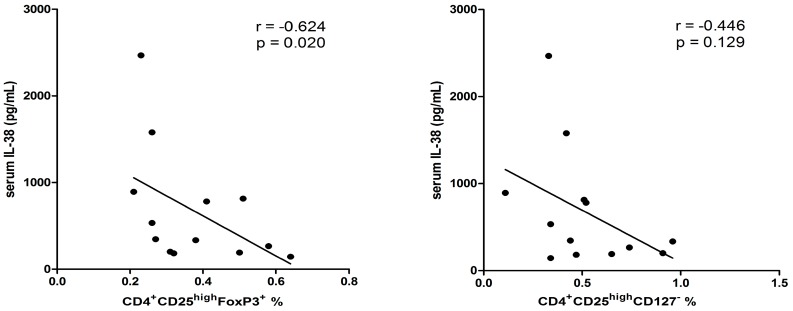
Correlations between serum IL-38 concentrations with peripheral regulatory T lymphocytes in asthmatic patients with high level of serum periostin (>40 ng/mL). *n* = 13. Correlation was determined by non-parametric Spearman’s correlation test.

Correlations of IL-38 with Treg cells, periostin, IL-36R and other asthma related cytokines and transcription factors were investigated. No obvious correlation was found between IL-38 and periostin (*p* = 0.190). As shown in Figure 4, in patients with higher periostin concentration (>40 ng/mL), serum concentration of IL-38 negatively correlated with the percentage of CD4^+^CD25^high^FoxP3^+^ (*r* = −0.624, *p* = 0.020) and CD4^+^CD25^+^CD127^−^ Treg cells (*r* = −0.446, *p* = 0.129). Since CD4^+^CD25^+^FoxP3^+^ and CD4^+^CD25^+^CD127^-^ Treg lymphocytes were analyzed by intracellular and surface staining, respectively, it may account for the difference of the correlation of these two types of Treg cells with IL-38 expression. The r value of correlation coefficient is expected to be higher when more severe asthmatic patients with higher level of periostin are recruited. Besides, as shown in Table 2, the mRNA level of IL-38 positively correlated with its receptor IL-36R and negatively correlated with IL-10 (*r* = 0.407, *p* = 0.006; *r* = −0.348, *p* = 0.019, respectively).

### 2.6. Determination of Activated CD4^+^CD25^+^CD134^+^ T Cells

**Figure 5:**
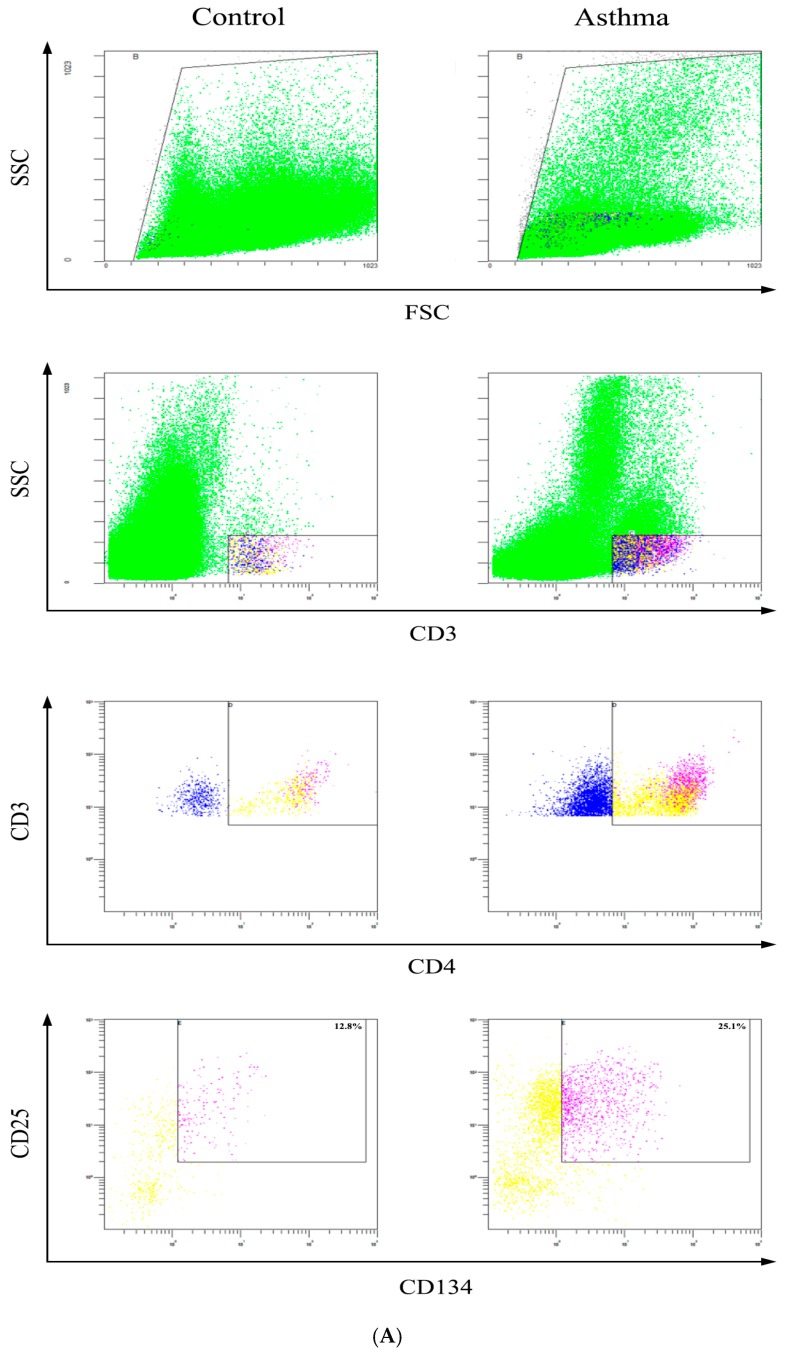

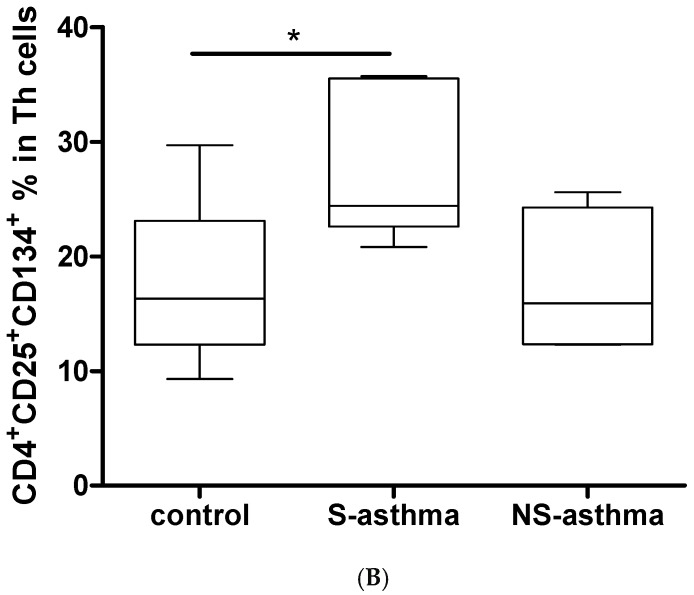
Determination of activated mitogen-specific CD4^+^CD25^+^CD134^+^ T cells from the in vitro whole blood in asthmatic patients and control. (**A**) The representative dot plot and gating strategy of activated CD4^+^CD25^+^CD134^+^ T cells in whole blood were shown; (**B**) The percentage of CD4^+^CD25^+^CD134^+^ T cells in asthmatic patients and control subjects was determined by flow cytometry (*n* = 8 in each group). Statistical significances were indicated by * *p* < 0.05 (Mann-Whitney U test). S-asthma: steroid-treated asthmatic patients; NS-asthma: non-steroid-treated asthmatic patients, control: control subjects.

It has been shown that CD134-CD134L interactions are crucial for the generation of memory T cells, promoting the survival of antigen-specific effector T cells [24]. Co-expression of T cell activation markers CD134 and CD25 surface molecules allows specific detection of activated antigen-specific CD4^+^ T cells following antigen/mitogen/allergen exposure. Highly activated CD4^+^CD25^+^CD134^+^ T cells therefore suggests that cells within this subset are inflammatory disease associated functional effector cells [25]. The representative dot plot and gating strategy of activated CD4^+^CD25^+^CD134^+^ T cells in whole blood were shown in Figure 5. After stimulation with T cell mitogen phytohaemagglutinin (PHA), the percentage of CD4^+^CD25^+^CD134^+^ T cells was significantly higher in whole blood from steroid treated asthmatic patients [24.4 (22.6–35.5) %] than that from control subjects [16.3 (12.3–23.1) %] (Figure 5), in the contrast with the down-regulated Treg cell subset. Although there is no significant increase in the percentage of CD4^+^CD25^+^CD134^+^ T cells in whole blood from steroid treated asthmatic patients in comparison with that of non-steroid treated patients, the median of mitogen activated T cells is higher in steroid treated asthmatic patients, probably because of their higher disease severity.

Dysregulation of cytokines and T cell functions are the immunological features in the pathogenesis of asthma [13,14]. The elevated anti-inflammatory Treg cell cytokines IL-10 and transforming growth factor (TGF)-β have been shown to be necessary for the induction of airway hypersensitiveness and airway remolding, respectively, in asthma [4,5,6]. Besides, the aberrant expression of the newly reported Breg and Treg cytokine IL-35 has also being involved in asthma [26]. However, the potential immunopathological roles of novel anti-inflammatory cytokine IL-38 in childhood asthmatic patients have never been extensively studied. This cross-sectional study demonstrated the up-regulation of anti-inflammatory cytokine IL-38 in asthmatic patients than controls (Figure 1).

Asthma has been well associated with Th2 cytokines IL-4, IL-5, IL-9 and IL-13 that play critical roles in orchestrating, perpetuating and amplifying the inflammatory response, although other cytokines, such as IL-6 and IL-1β, are involved in many inflammatory diseases [27,28,29]. In the present study, the elevated serum Th2 cytokines IL-5 and IL-13 levels have also been observed in asthmatic patients although IL-4 was undetectable (Figure 1). Other pro-inflammatory cytokines IL-17, IL-6, IFN-γ and IL-1β were also increased in patients with asthma compared with control subjects (Figure 1). IL-38 is a newly named IL-1 cytokine family member and functions similar with IL-36Ra [15,17]. Other pro-inflammatory cytokines IL-17, IL-6, IFN-γ and IL-1β were also increased in patients with asthma compared with control subjects (Figure 1). IL-38 is a newly named IL-1 cytokine family member and functions similar with IL-36Ra [15,17].

Currently, the expression and function of IL-38 in childhood asthma remains unclear although IL-36α and IL-36γ were suggested to be involved in the regulation of neutrophilic airway inflammation in asthma [30,31]. In the present cross sectional study, the elevated serum concentration of IL-38 in asthmatic patients was observed for the first time. The mRNA level of IL-38 was also overexpressed in patients although the elevation did not reach the significance (Figure 3). However, the negative correlation of IL-38 with IL-10 was observed in all asthmatic children (Table 2). During the exacerbation of asthmatic inflammatory condition, it is reasonable that both anti-inflammatory and pro-inflammatory cytokines can be increased because the inflammatory cytokines-mediated inflammation can trigger the release of the regulatory cytokines including anti-inflammatory IL-10, IL-35 and IL-38. However, the elevated IL-38 and other regulatory/anti-inflammatory cytokines may not be sufficient to down-regulate the elevated level of pro-inflammatory cytokines IL-17, IL-6, IFN-γ and IL-1β [26].

Imbalance of the functions of Th17 and Treg cells also plays crucial roles in the pathological mechanism in asthma. In this study cohort, serum IL-17A concentrations were found to be significantly higher in asthmatic patients without or with steroid treatment comparing with control group (Figure 1) which is in concordance with our previously published results for the activation of Th17 cells in asthma [13,32]. The Th2 and Th17-mediated immunity is normally suppressed by Treg cells, which are essential in the maintenance of immunological tolerance to “self” and in the regulation of the immune response against infectious organisms, both pathogens and commensals. Tao et al. reported that the frequency of Th17 cells, Th17 transcription factor ROR-γt mRNA expression, and the plasma concentration of IL-17 were significantly higher, while Treg cells and TGF-β1 were significantly lower in children with allergic rhinitis (AR) accompanying with bronchial asthma (BA), compared with those in children with AR or BA alone or control subjects. This is consistent with the present study of the declined circulating CD4^+^CD25^high^FoxP3^+^ and CD4^+^CD25^high^CD127^−^ Treg frequencies in asthmatic children with or with steroids treatment. Moreover, the elevated circulating anti-inflammatory IL-38 concentration correlated negatively with Treg subsets in patients having higher level of periostin, which is an eosinophilic airway inflammation biomarker (Figure 4). Thus, IL-38 may have an inter-relationship with suppressive Treg cells during the process of asthma. However, the mechanism of how IL-38 modulates Treg cells and immune response in asthma requires further investigation. A distinct subtype of asthma was defined by the expression of genes inducible by Th2 cytokines in bronchial epithelium. However, identification of this subtype depends on invasive airway sampling. Periostin is a newly found circulating biomarkers of airway eosinophilia in asthmatic patients [33]. As expected, this cross-sectional study demonstrated the up-regulation of periostin in more severe (steroid treated) asthmatic children patients than controls. Besides, the percentage of activated CD4^+^CD25^+^CD134^+^ T lymphocytes in whole blood culture was significantly higher in patients with asthma (Figure 5).

## 3. Experimental Section

### 3.1. Ethics Statement

The protocol was approved by the Clinical Research Ethics Committee of the Chinese University of Hong Kong-New Territories East Cluster Hospitals, and informed written consent was obtained from all subjects or their parents in accordance with the 1964 Declaration of Helsinki and its later amendments.

### 3.2. Asthmatic Patients, Non-Allergic Control Subjects and Blood Samples

Forty primary school children aged 6 to 8 years with asthma were recruited in Hong Kong. The diagnosis of asthma was made according to the International Study of Asthma and Allergies in Childhood (ISAAC) questionnaire data. Anti-asthma treatments in our patient cohort were stable for at least 4 weeks before study. Twenty age and sex-matched non-allergic control subjects were recruited. None of them have any history of allergic or inflammatory disease and did not require any drug treatment at the time of study. All patients and control subjects were recruited by clinicians in Department of Pediatrics, Prince of Wales Hospital, Hong Kong. All subjects were free from respiratory tract infection, asthma exacerbation, and the use of systemic corticosteroids for 4 weeks prior to study. The atopic status of these patients and control subjects was ascertained by positive serum-specific IgE assays to house dust mite Dp1 and Df1, cat dander and cockroaches by fluorescence enzyme immunoassay (AutoCAP analyzer, Pharmacia Diagnostics AB, Uppsala, Sweden). Ten milliliters (mL) of EDTA-anti-coagulated blood were collected from each patient and control subject. Plasma and serum samples were preserved at −80 °C for subsequent assays. All the enrolled subjects were ethnic Chinese.

### 3.3. Quantitative Analysis of Cytokines and Periostin

Serum concentrations of IL-38 and periostin were measured using enzyme-linked immunosorbent assay (ELISA) reagents from Adipogen (Adipogen International, San Diego, CA, USA) and Raybiotec (Norcross, GA, USA), respectively. Serum and supernatant IL-5, IL-13, IL-17, IFN-γ, IL-6 and IL-1β were determined by using multiplex assay kit reagents from Merck Millipore (Billerica, MA, USA) using Bio-plex 200 suspension array system (Bio-Rad Laboratories, Hercules, CA, USA).

### 3.4. Flow Cytometry of Treg Lymphocytes

PBMC from asthma patients and controls were purified using Ficoll Plus gradient centrifugation (GE Healthcare Bio-Sciences, Piscataway, NJ, USA) from EDTA venous peripheral blood (13 mL). The plate was pre-treated with anti-CD3 (3 μg/mL) for 2 h at 37 °C in a 5% CO_2_ atmosphere and washed three times with PBS. Then PBMC (5 × 10^6^/mL/well) in RPMI with 10% inactivated FBS were seeded and incubated with anti-CD28 (1 μg/mL) for 24 h at 37 °C in a 5% CO_2_ atmosphere. The culture medium used was free of detectable endotoxin (<0.1 EU/mL). Harvested cells were stained with rat FITC-conjugated anti-human CD4, PC5-conjugated anti-human CD25, PE-conjugated anti-human CD127 and their corresponding isotype control antibodies (eBioscience, San Diego, CA, USA). For intracellular staining, cells stained with CD4 and CD25 were fixed and Fc receptors were blocked with normal rat serum, and then incubated with PE-conjugated anti-human FoxP3 (eBioscience) in permeabilization buffer at 4 °C for 45 min in the dark. After washing, the proportion of CD4^+^CD25^high^CD127^−^ and CD4^+^CD25^high^FoxP3^+^ Treg lymphocytes was quantified using FACSAria flow cytometer (BD Pharmingen Corp., San Diego, CA, USA).

### 3.5. Real time PCR

Total RNA of human PBMCs (1 × 10^6^ cells) were extracted and transcribed into first-strand complementary DNA using PrimeScript™ RT Master Mix (Takara Bio Inc., Shiga, Japan). The quantitative expression of target genes was measured using FastStart Universal SYBR Green Master (ROX) (Roche Applied Science, Basel, Switzerland) with corresponding primers (Table 3). The GAPDH house keeping gene was used as the internal reference and the relative gene expression was calculated using 2^−ΔCt (Ct, target gene−Ct, GAPDH)^.

### 3.6. Detection of Activated CD4+ T Cells

The percentage of CD25^+^CD134^+^ activated CD4^+^ T cells in whole blood of asthma patients and controls were determined by Act-T4 Cell™ Kit (Cytognos, Salamanca, Spain). Briefly, 0.5 mL of sodium heparin-anti-coagulated whole blood was mixed with 0.5 mL of culture medium (RPMI-1640 supplemented with 2 mM l-glutamine, 100 U/mL penicillin and 100 μg/mL streptomycin) in sterile well cell culture plate. For each sample to study, negative control culture and positive control culture with PHA (5 μg/mL) were prepared. Then all cultures were incubated at 37 °C for 44–48 h in a humidified atmosphere of 5% CO_2_ in air. After incubation, 100 μL of each culture (negative control and positive control) was pipetted into separated FACS tubes and 20 μL of the 4-antibody mixture (CD3, CD4, CD25 and CD134) provided in the Act-T4 Cell™ kit was added to each tube. After mixing and incubating for 15 min at room temperature, 1 mL of erythrocyte lyse-non-wash solution was added and incubated for 10 min at room temperature protected from light. Then the cells were analyzed by Navios flow cytometer (Beckman Coulter Inc., Miami, FL, USA).

### 3.7. Statistical Analysis

Results were expressed as mean ± SD, or median (interquartile range, IQR) if data were not normally distributed. All statistical analysis was performed by the SPSS statistical software for Windows, version 10.1.4 (SPSS, Inc., Chicago, IL, USA). For continuous variables, statistical significance was calculated using Mann-Whitney U test. Non-parametric spearman’s test was used to assess the correlation of two variables. All hypotheses were 2-tailed, and *p* values < 0.05 were considered to be significant.

## 4. Conclusions 

Together, this clinical study demonstrates the elevation of anti-inflammatory cytokine IL-38 and the decrement of CD4^+^CD25^high^FoxP3^+^ and CD4^+^CD25^high^CD127^−^ Treg in childhood asthma. The negative correlation of IL-38 and Treg lymphocytes may imply a negative feedback of the two anti-inflammatory factors in asthma. The detailed intercellular mechanism by which IL-38 modulates Treg cells for the suppression of inflammation in asthma requires further investigations.

## Figures and Tables

**Table 1:** Demographic, clinical and laboratory data of the asthmatic patients with or without steroid treatment and control subjects.

Characteristics of Subjects	Non-Steroid-Treated Patients (*n* = 20)	Steroid-Treated Patients (*n* = 20)	Control Subjects (*n* = 20)
Sex (male/female)	9/11	8/12	10/10
Age, year (mean ± SD, range)	7.3 ± 0.6, 6–9	7.5 ± 0.6, 6–9	7.8 ± 0.9
Eosinophil % in PB	5.0 (4.0–6.0) ***	5.5 (5.0–6.0) ***	2.0 (1.0–2.0)
Basophil % in PB	1.0 (0.0–1.0)	1.0 (1.0–1.0)	1.0 (1.0–1.0)
Presence of at least one			
Positive allergen-specific IgE, n (%)	18 (90%)	19 (95%)	0 (0)
House dust mite Dp1, n (%)	12 (60%)	13 (65%)	0 (0)
House dust mite Df1, n (%)	11 (55%)	10 (50%)	0 (0)
Cockroaches, n (%)	8 (40%)	7 (35%)	0 (0)
Cat dander, n (%)	4 (20%)	5 (25%)	0 (0)

Values were given as median (IQR) or mean ± SD; PB: peripheral blood; *** *p* < 0.001 comparing with controls.

**Table 2:** Correlations between mRNA levels of IL-38 with IL-36R, IL-13, IL-10 and FoxP3 in all asthmatic patients with or without steroid treatment. Correlation was determined by non-parametric Spearman’s correlation test.

mRNA Expression	IL-38 mRNA
IL-36R mRNA	*r* = 0.407, *p* = 0.006 **
IL-13 mRNA	*r* = 0.156, *p* = 0.714
IL-10 mRNA	*r* = −0.348, *p* = 0.019 *
FoxP3 mRNA	*r* = −0.093, *p* = 0.566

* *p* < 0.05; ** *p* < 0.01.

**Table 3:** Primers for different human genes used in real time PCR.

Primer		Sequence of Primers
IL-10	Forward	5′-GCCTAACATGCTTCGAGATC-3′
	Reverse	5′-TGATGTCTGGGTCTTGGTTC-3′
IL-36R	Forward	5′-GCTGGAGTGTCCACAGCATA-3′
	Reverse	5′-GCGATAAGCCCTCCTATCAA-3′
IL-38	Forward	5′-TTATCCTTGTGGGCTCAGTT-3′
	Reverse	5′-AATCCGTTCCCTTGGCTTTT-3′
IL-13	Forward	5′-TGAGGAGCTGGTCAACATCA-3′
	Reverse	5′-CAGGTTGATGCTCCATACCAT-3′
FoxP3	Forward	5′-GCACATTCCCAGAGTTCCT-3′
	Reverse	5′-TTGAGTGTCCGCTGCTTC-3′
GAPDH	Forward	5′-ATGGGGAAGGTGAAGGTCG-3′
	Reverse	5′-GGGGTCATTGATGGCAACAATA-3′
